# 4211 Longitudinal cohort study of the association between atopic dermatitis and depression/anxiety throughout childhood

**DOI:** 10.1017/cts.2020.133

**Published:** 2020-07-29

**Authors:** Chloe E Kern, Kaja Lewinn, Joy Wan, Katrina Abuabara

**Affiliations:** 1 University Of California, San Francisco

## Abstract

OBJECTIVES/GOALS: Atopic dermatitis is one of the most common chronic childhood conditions worldwide and is associated with poor mental health outcomes. Our aim is to determine whether childhood atopic dermatitis is associated with symptoms of depression throughout childhood and adolescence, and whether this association is mediated by serum inflammatory markers. METHODS/STUDY POPULATION: We will perform a longitudinal analysis of over 7000 children from an existing prospective cohort. The primary exposure is atopic dermatitis (AD) annual period prevalence measured by a standardized questionnaire at 12 time points between age 6 months and 16 years. Depression is measured using self-reported responses to the Short Moods and Feelings Questionnaire at 6 time points between 10 and 18 years of age. Cross-sectional regression analyses will be performed to compare depressive signs between children with and without AD and test for dose-response effects with AD and depression. Longitudinal analyses will be conducted using mixed-effects models to estimate the average effect across childhood. We will complete a mediation analysis to determine the extent to which IL-6 and CRP mediate this association. RESULTS/ANTICIPATED RESULTS: We anticipate that atopic dermatitis will be associated with SMFQ scores in a dose response relationship, and that inflammatory markers CRP and IL-6 will partly mediate this association. DISCUSSION/SIGNIFICANCE OF IMPACT: Childhood is a critical time for mental health. Understanding the longitudinal relationship between atopic dermatitis, depression, and inflammatory mediators is crucial as new biologic treatments targeting inflammatory cascades are approved for atopic dermatitis and have the potential to prevent mental health conditions.

